# Application of toll-like receptors (TLRs) and their agonists in cancer vaccines and immunotherapy

**DOI:** 10.3389/fimmu.2023.1227833

**Published:** 2023-10-23

**Authors:** Samik Chakraborty, Juan Ye, Herui Wang, Mitchell Sun, Yaping Zhang, Xueyu Sang, Zhengping Zhuang

**Affiliations:** ^1^ Neuro-Oncology Branch, National Cancer Institute, National Institutes of Health, Bethesda, MD, United States; ^2^ NE1 Inc., New York, NY, United States

**Keywords:** TLR - toll-like receptor, TLR agonists, cancer vaccine, immunotherapy, adjuvant, cancer immuno therapy

## Abstract

Toll-like receptors (TLRs) are pattern recognition receptors (PRRs) expressed in various immune cell types and perform multiple purposes and duties involved in the induction of innate and adaptive immunity. Their capability to propagate immunity makes them attractive targets for the expansion of numerous immunotherapeutic approaches targeting cancer. These immunotherapeutic strategies include using TLR ligands/agonists as monotherapy or combined therapeutic strategies. Several TLR agonists have demonstrated significant efficacy in advanced clinical trials. In recent years, multiple reports established the applicability of TLR agonists as adjuvants to chemotherapeutic drugs, radiation, and immunotherapies, including cancer vaccines. Cancer vaccines are a relatively novel approach in the field of cancer immunotherapy and are currently under extensive evaluation for treating different cancers. In the present review, we tried to deliver an inclusive discussion of the significant TLR agonists and discussed their application and challenges to their incorporation into cancer immunotherapy approaches, particularly highlighting the usage of TLR agonists as functional adjuvants to cancer vaccines. Finally, we present the translational potential of rWTC-MBTA vaccination [irradiated whole tumor cells (rWTC) pulsed with phagocytic agonists Mannan-BAM, TLR ligands, and anti-CD40 agonisticAntibody], an autologous cancer vaccine leveraging membrane-bound Mannan-BAM, and the immune-inducing prowess of TLR agonists as a probable immunotherapy in multiple cancer types.

## Introduction

Emerging strategies in cancer immunotherapy, including immune checkpoint inhibitors (ICIs), cancer vaccines, and chimeric antigen receptor-T cells (CAR-T), have shown exceptional promise in clinical trials, giving rise to a plethora of ongoing research and development in this field (1). ICIs, including antibodies targeting anti-CTLA4 and anti-PD-1/PDL-1, have significantly progressed in various clinical trials to treat diverse cancer types (1). Meanwhile, adoptive cell therapies like CAR-T have shown promising results in treating multiple hematopoietic malignancies (2). Additionally, regulatory agencies have approved several preventive and therapeutic cancer vaccines for treating different cancers with numerous other vaccines in various development stages (3–7). These immunotherapies have underscored the importance of stimulating a robust anti-tumor immune response in cancer patients as a potential avenue for combating cancer. Exploring and harnessing appropriate immunostimulatory mechanisms is crucial in developing new cancer immunotherapy approaches. Toll-like receptors (TLRs) are a particular group of membrane receptor molecules that play the above-mentioned immunostimulatory functions in several innate immunity pathways (8). Consequently, TLRs are some of the most sought-after molecules used as vaccine adjuvants and in several immunotherapeutic approaches related to preventing and treating several infectious diseases, including cancer (8).

The inception of cancer immunotherapy dates back over a century, with the initial attempts involving using bacteria or bacterial products to activate the immune system (9–11). In 1891, William Coley pioneered the field of immunotherapy by administering a blend of heat-inactivated *Streptococcus pyogenes* (a Gram-positive bacteria) and *Serratia marcescens* (a Gram-negative bacteria) through intratumoral injections (9–11). This bacterial mixture became known as Coley’s toxin later (11, 12). This approach by Coley achieved a robust immune response against sarcomas, resulting in reduced tumor growth and, in some cases, tumor elimination even though the inherent mechanism remained unclear at that time (10–12). Subsequent research elucidated this therapeutic response, unveiling the significance of unique signaling molecules like pattern recognition receptors (PRRs) and pathogen-associated molecular patterns (PAMPs), which serve as ligands or activators for PRRs (11, 13–15).

**Figure 1:**
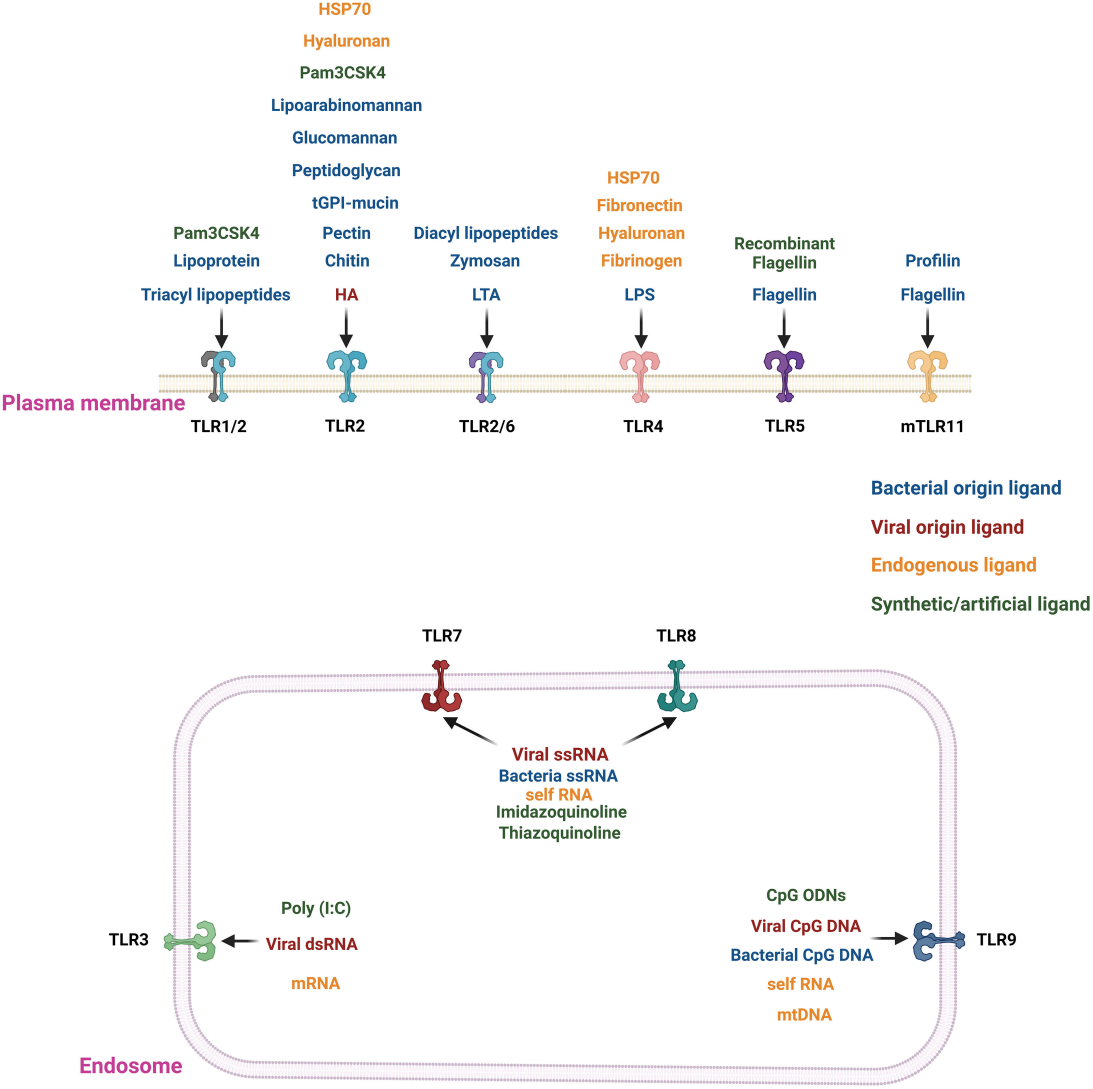
Location and agonists of different TLRs. TLRs 1/2, 2, 2/6, 4, and 5 are present in the extracellular region of the plasma membrane. In contrast, TLRs 3, 7, 8(only in humans), and 9 are localized on the endosomal membrane. The TLRs are stimulated by their specific ligand or agonists. The extracellular TLRs are mainly activated by exogenous agonists of bacterial, viral or pathogenic origin. The major exogenous ligands or microbial agonists are flagellin protein from bacterial flagella, lipoteichoic acid (LTA) and peptidoglycan (PGN) from Gram-positive bacteria, LPS from Gram-negative bacteria, lipoarabinomannan (LAM), lipopeptides, lipoglycans, and lipomannans from mycobacteria, zymosan from yeast (20, 21). There are synthetic TLR agonists like Pam3CSK4 or recombinant flagellin to stimulate the extracellular TLRs (20, 21). In contrast, nucleic acid ligands stimulate the endosomal TLRs 3, 7, 8, and 9. For example, TLR3 is stimulated by viral double-stranded RNA (dsRNA); TLR7 and 8 are triggered by viral and bacterial single-stranded RNA (ssRNA), and TLR9 recognizes CpG DNA from viruses and bacteria (20–25). There are also some endogenous ligands resulting from cellular injury, cell death, extracellular matrix components (e.g., hyaluronan, fibronectin, and fibrinogen), plasma membrane constituents, nuclear and cytosolic proteins and heat shock proteins, and elements of damaged/fragmented organelles such as mitochondrial DNA (mtDNA) (26, 27). TLRs 2 and 4 bind to most of the endogenous ligands resulting from cellular injury, cell death, and extracellular matrix components (27). The endogenous nucleic acid ligands like RNA or mtDNA bind with the endosomal ligands TLRs 3, 7, 8, and 9 (26, 27).

TLRs are a subclass of receptors from the PRR family and serve as central players in innate immune responses (16). TLRs are transmembrane domain proteins (type I) with tripartite motifs (16, 17). TLRs feature three distinct functional domains: an leucine-rich repeats (LRRs) containing amino (N)-terminal responsible for ligand binding (folded into a typical horseshoe-like structure), a transmembrane spanning region, and a carboxyl (C)-terminal cytoplasmic domain resembling the cytoplasmic region of globular Toll/interleukin-1 (IL-1) receptor (TIR) (16, 17). To date, ten human and thirteen murine TLRs have been identified (18). Based on their subcellular localization, TLRs are categorized into extracellular and intracellular groups (16, 19). TLRs such as TLR1, TLR2, TLR5, TLR6, and TLR10 are exclusively expressed on the plasma membrane and belong to the extracellular group; while TLR3, TLR7, TLR8, and TLR9 fall within the intracellular group, are expressed on the endosome and endoplasmic reticulum (16, 19). Only TLR4 is present in both intracellular components and the plasma membrane (16, 19). For each of the TLRs, there is a specific ligand(s) (**Figure 1**, **Table 1**). Every TLR with its ligand activates specific downstream signaling pathways either through myeloid differentiation primary response protein 88 (MyD88) and/or TIR-domain-containing adapter-inducing IFNβ (TRIF) (16, 20, 39–41). TLR-mediated signaling initiates the secretion of multiple cytokines that enhance the immune system’s ability to combat external pathogens and infectious agents (16, 17, 39). Moreover, TLRs play a crucial role in activating and maturing various immune cells involved in innate and adaptive immune responses (16–18). We have listed the location of all the TLRs and their agonists in **Figure 1**. Moreover, we have provided a detailed classification, localization, and involved ligands of the TLRs in **Table 1**.

**Table 1:** Expression, localization, agonists/ligands of different TLRs.

TLR	Species	Localization	Microbial ligands	Microbes expressing TLR ligands	Endogenous ligands	Synthetic agonists	Ref.
TLR1	Human and mouse	Plasma membrane	Triacyl lipopeptides Bacterial lipoprotein	*Mycobacterium tuberculosis*	Unknown	Pam3CSK4	(20, 28)
TLR2	Human and mouse	Plasma membrane	Lipoproteins, zymosan, lipoarabinomannan, peptidoglycan, lipoteichoic acid	*Mycoplasma, Neisseria meningitides, Haemophilus influenzae, Leishmania major*, *Staphylococcus aureus*, Herpes simplex virus, Measles virus	Versican	Pam2CSK4, Pam3CSK4	(20, 28–32)
TLR3	Human and mouse	Endosome	Viral dsRNA	Reovirus	mRNA	Poly(I:C), poly-ICLC, poly(I:C_12_U) poly(A:U)	(20, 21, 33)
TLR4	Human and mouse	Plasma membrane and Endosome	LPS	*Escherichia coli, Pseudomonas aeruginosa*	Oxidized low-density lipoprotein, Amyloid-beta protein	Monophosphoryl lipid-A (MPL) derivatives	(20, 21, 33, 34)
TLR5	Human and mouse	Plasma membrane	Flagellin	*Salmonella* sp.	Unknown	Recombinant flagellin derivatives	(20, 21, 33)
TLR6	Human and mouse	Plasma membrane	Diacyl lipopeptides, lipoteichoic acid, zymosan	*Mycoplasma*, Hepatitis C virus (HCV), Cytomegalovirus	Oxidized low-density lipoprotein, Amyloid-beta protein, versican	Macrophage-activating lipopeptide 2, synthetic diacylated lipoproteins, Pam2CSK4	(20, 21, 29)
TLR7	Human and mouse	Endosome	Viral and bacterial ssRNA	Human immunodeficiency virus (HIV), HCV	Immune complexes, self-RNA	Thiazoquinoline and imidazoquinoline derivatives (e.g., resiquimod, imiquimod)	(20, 21, 33) (35, 36),
TLR8	Human and mouse	Endosome	Viral and bacterial ssRNA	Human immunodeficiency virus (HIV), HCV	Immune complexes, self-RNA	Thiazoquinoline and imidazoquinoline derivatives (e.g., resiquimod, imiquimod)	(20, 21, 33) (35, 36),
TLR9	Human and mouse	Endosome	Viral and bacterial CpG DNA, DNA: RNA hybrids	Human papilloma virus (HPV), Hepatitis B virus (HBV), Epstein-Barr virus (EBV), Polyomavirus	Chromatin-IgG-immune complexes, self-DNA	CpG Oligodeoxynucleotides (CpG ODNs)	(20, 33, 37)
TLR10	Human	Plasma membrane	Unknown		Unknown	Unknown	(20)
TLR11	Mouse	Endosome	Profilin and flagellin	*Toxoplasma gondii*	Unknown	Unknown	(38)
TLR12	Mouse	Endosome	Profilin		Unknown	Unknown	(20)
TLR13	Mouse	Endosome	Bacterial 23S ribosomal RNA (rRNA)		Unknown	23S rRNA-derived oligoribonucleotide	(20)

Adapted from “Toll-like receptors: Activation, signalling and transcriptional modulation.” by De Nardo D. 2015, Cytokine. 74(2):181-9.

This review aims to consolidate the current strategies involving TLR agonists as potential therapeutics for cancer, either as standalone treatments, in combination therapies or as adjuvants for cancer vaccines. We also explore the supporting evidence for TLR agonists as adjuvants in cancer vaccines, promoting innate and adaptive immune responses against cancer cells, specifically focusing on the rWTC-MBTA autologous vaccine developed by our research group.

## The roles of TLRs in various immune cell types and their impact on the regulation of cancer

It’s crucial to note that TLRs are present in various types of cells, encompassing innate immune system components such as macrophages, neutrophils, dendritic cells (DCs), natural killer (NK) cells, and mast cells. They are also found in adaptive immune system elements like T and B lymphocytes, stromal cells, and various tumor cells. When TLRs engage with a ligand, they can significantly boost the expression of multiple costimulatory molecules on the cell membranes, activating cytokines and T-cell activation (16, 17). The following section briefly discusses how TLRs stimulate different immune cells and their role in regulating cancer immunity.

### Dendritic cells

Dendritic cells (DCs) are widely recognized as the immune system’s most efficient professional antigen-presenting cells (APCs) (42). DCs are also the most studied cells among all the TLR-expressing immune cells in the milieu of instigation of adaptive immunity (42). When faced with an infection or inflammation, immature DCs undergo a process of activation and transformation into mature DCs. These mature DCs are responsible for activating adaptive immune cells such as B and T lymphocytes (43). The maturation of DCs involves a series of complex stages, including changes in the composition of receptors involved in endocytosis and phagocytosis, increased expression of co-stimulatory molecules like CD40, CD58, and CD86, alterations in morphology, and reorganization of lysosomal and MHC compartments (44). It’s important to note that the DC population is highly diverse, consisting of various subtypes that exhibit differences in their functions, phenotypes, and distribution within the body (45). The two central populations of DCs found in the human immune system are lymphoid‐derived plasmacytoid dendritic cells (pDCs) and myeloid‐derived dendritic cells (mDCs) (45). Both pDCs and mDCs can activate the CD4^+^ and CD8^+^ T cells as well as facilitate the process of antigen cross-presentation to initiate the proliferation of CD8^+^ T cells (46–48). Other phenotypes of DCs are monocyte-derived DCs (moDCs) and CD34+ cell–derived DCs (49). The activation status of dendritic cells is critical in determining how the immune system responds to a particular threat. All the subsets of DCs express distinctive TLRs, permitting themselves to generate a dedicated response against different pathogens (44). The major TLRs involved in DC maturation and function are TLR2, 3, 4, 5, 7/8, and 9 (50). TLR signaling is the key to DC-mediated cytotoxic T-cell activation (50). Previous studies demonstrated that TLR-mediated stimulation augments maturation and antigen presentation of murine DCs followed by induction of cytotoxic T cells (51, 52). Maturation of DC sub-populations and onset of cytotoxic CD8^+^ T cells through IL-27-mediated signaling were reported after ligand mediated activation of TLR3 and TLR7 (51, 52). As suggested by the distinct Toll-like receptor (TLR) expression profiles in different DC subsets, the pDCs are primarily activated by viral pathogens, while mDCs primarily respond to fungal and bacterial antigens, and these functional characteristics are exploited in DC-mediated vaccination and immunotherapy (53, 54). Similarly, the anti-tumor outcome of TLR7 activation was evident in central nervous system tumors, increasing maturation of DCs and activating tumor specific cytotoxic CD8^+^ T cells (55). Another report demonstrated TLR mediated enrichment of the Th1 microenvironment and promoted activation of cytotoxic T-cells via IFN-λ-induced IL-12 released by breast cancer-associated dendritic cells (56). TLR4 activation also induced anti-colorectal cancer T cell response *in vitro* through DC maturation (57). Likewise, activation of TLR-4 and processing of tumor antigens stimulate DC maturation to markedly increasing *in vivo* CD8^+^IFNγ^+^ cytotoxic T cells (58). Interestingly, activating TLR7 and 8 was a crucial step in promoting the maturation of DCs isolated from AML patients and subsequently activating cytotoxic T cells *in vitro* (59). Interestingly, TLR7 signaling activated plasmacytoid dendritic cells (pDCs) leading to killing of murine melanoma cells through stimulation of NK cells and activating CD8^+^ cytotoxic T-cells (60). Similarly, in ALL patients, activation of pDCs vial TLR9 molecules led to increased IFN production, which stimulated NK cells through TRAIL and CD69-mediated signaling (61). An earlier report about DC-targeted vaccines, demonstrated CD8^+^ T cell response and better therapeutic efficacy after the activation of DCs through TLR7/8 and TLR3 mediated signaling (62). The immunosuppression present in the tumor microenvironment (TME) impedes the success of cancer immunotherapy, and DCs are extremely important in generating anti-tumor immunity inside the TME (42). Research has shown that the tumor microenvironment (TME) can hinder the growth and differentiation of mDCs (63). However, multiple studies demonstrated that TLR-mediated signaling has the potential to reactivate the immune functions of these inhibited dendritic cells, and this method could be beneficial to effectively counteract immunosuppression in the TME, offering a new arrow in the quiver of immunotherapy (64, 65).

### Macrophages

Macrophages were the first immune cells identified to uphold tissue homeostasis, facilitate tissue repair, orchestrate immune responses, and combat pathogens (66, 67). Subsequently, it became evident that they also infiltrate and inhabit tumor sites and influence tumor development (66, 67). In addition, macrophages can alter their transcriptional profile, display remarkable cellular plasticity, and modify their functions in response to various inflammatory, tissue-specific, external pathogenic, and environmental stimuli, leading to anti-tumor and pro-tumor effects (67). In inflammatory conditions, classifying the tumor-associated macrophages (TAM) is still a complex task (66, 67). Classically, the macrophages were mainly categorized into two polarization states: M1, with pro-inflammatory traits, and M2, with anti-inflammatory characteristics (66, 67). M2 macrophages were considered to support tissue remodeling, tumor growth, and cancer-related processes, including cell proliferation, invasion, metastasis, and immune suppression; whereas, M1 macrophages were designated to drive immune responses, cause tissue damage, and inhibit tumor growth by enhancing anti-tumor responses of T cells and natural killer cells (66, 67). Macrophages are one of the most influential players in the TME, rendering them as a vital point for cancer immunotherapy (68). The anti-inflammatory M2 subtype of macrophages supports tumor growth and maintenance, but the pro-inflammatory M1 subtype promotes inflammation and tumoricidal properties (68). Switching the M2 subtypes to M1 in the tumor microenvironment by stimulants can promote tumoricidal activity (69). Regrettably, this simplified classification of macrophages into just the M1 or M2 category failed to define the diverse range of macrophage polarization states present within tumors or the TME (70), leading to a modern classification of TAMs where M1 and M2 represent the extremes of a spectrum with numerous intermediate subsets (66, 67). In relation to the complex interactions between different cell types within the TME, the TAMs are now considered into two main subtypes, M1-like (pro-inflammatory macrophages) and M2-like (anti-inflammatory macrophages) (67). Stimulation of Toll-like receptors (TLRs) in macrophages has been long recognized as a mechanism that drives macrophages toward a pro-inflammatory phenotype, and this renders TLR agonists particularly attractive in the context of cancer immunotherapy (71). It’s noteworthy that since 2015, over 60 clinical trials have been initiated to assess the therapeutic potential of TLR agonists in treating various cancers (4). Activation of TLR3, recruits *in vitro* and *in vivo* IFN signaling cascade resulting in switching to M1 phenotype from M2 phenotype (69). The switching of M2 to M1 involves signaling associated with CD86, CD80, CD40, IL-12, IL-6, and TNF-α ensuing in enhanced antigen uptake by the macrophages and activation of T cells-mediated mice tumor growth regulation (72). Comparable anti-tumor results were detected in mice models of Lewis lung carcinoma and sarcoma following induction of TLR3 and TLR4 mediated signaling, respectively (73, 74). TLR4 was also suspected to promote the migration of macrophages through the upregulation of proinflammatory molecules like TNF-α, NF-κB, and VEGF (75). In a similar study, TLR-mediated signaling promoted the antitumor M1 phenotypes along with the upregulation of immunostimulatory cytokines like IL-18 (76). These immunostimulatory cytokines directed an antitumor collaboration between macrophages and NK cells *ex vivo* in ovarian cancer to stimulate IFN-γ secretion and Th1-type immune responses via NK cells (76). It is worth noting that TLR7/8 activation has been found to influence the differentiation of myeloid-derived suppressor cells (MDSCs) towards M1 phenotype within the tumor microenvironment, ultimately resulting in a regression of colorectal tumors in mice and a decrease in resistance to oxaliplatin (77). Oxaliplatin hindered the transformation of MDSCs into M1-like macrophages, but in combination with TLRs 7/8 agonist R-848, this hindrance was overcome (77). The addition of R-848 augmented the polarization of MDSCs to M1-like pro-inflammatory macrophages leading to increased apoptosis of the colorectal tumor cells (77). Furthermore, the stimulation of macrophages by TLR2/6 led to the activation of the NK cells and cytotoxic CD8^+^-T cells in several tumors, including metastasis mice models and pancreatic cancer (78, 79). This was accompanied by increased immune surveillance in tumors with concomitant increase of COX-2 expression in macrophages (78). COX-2 is the rate limiting enzyme of Prostaglandin E2 (PGE2) biosynthesis, and PGE2 is a strong suppressor of NK cells in the TME (78). Macrophage-activating lipopeptide-2 (MALP-2), a TLR2/6 agonist, enhances NK cell cytotoxicity towards the tumor cells, while the PGE2 mediated immunosuppression was blocked by COX-2 inhibitor (78).

### NK cells

NK or natural killer cells are a group of lymphocyte, an indispensable component of the innate immune system, and they are best recognized for killing pathogen or virus infected cells and also responsible to detect and regulating initial signs of cancerous tissues (80). NK cells are termed as the first rank of defense against cancer cells, with the capability to kill the cancer cells without any prior activation or priming. That is why they are named “natural killers” (80). Multiple reports documented that depending upon originating population, NK cells express almost all types of TLRs (81). Amid all the TLR ligands TLR3, 7, 8, and 9 mediated signaling demonstrated a significant role in cancer biology. Human NK cell lines for instance YTC12, YTS, and NK92 expressed high amounts of activated TLR3, causing cytotoxic killing effects on K562 cancer cells (82). Furthermore, head and neck squamous cell carcinoma (HNSCC) cells are killed via IFNγ secreting NK cells activated through TLR3 (82, 83). TLRs like TLR7, 8, and 9 can sense foreign nucleic acids, and their subsequent activation on NK cells empowers anti-tumor immune responses (84, 85). Though the activation of this nucleic acid-sensing, NK cell-associated TLRs are mostly dependent on the signaling induced by other cells present in the associated tumor microenvironment (84, 85). While there is some argument concerning the expression of TLR7 and 8 on NK cells (84, 85), several reports depicted the activation and proliferation of NK cells by the cytokines secreted from neighboring cells of the tumor microenvironment (84, 85). In addition to TLR9 stimulated cytotoxicity of NK cells on B16 melanoma cells, the secretion of inflammatory cytokines as IFNγ and IL-12 was promoted via TLR7/8 activation, which in order aided the NK cells to eliminate the HNSCC cells and B16-F10 melanoma cancer cells (86–88). Interestingly, another report showed an acceleration of antitumor activity of HER2(human epidermal growth factor receptor 2)-targeting monoclonal antibodies both *in vivo* and *in vitro* after TLR2-mediated activation of NK cells (89).

### B cells

B cells are the production house of antigen-specific antibodies and considered as the epicenter of the adaptive immune system (90). To date, several distinct B-cell subsets have been identified performing diverse functions in both adaptive and innate immune responses (90). B cells express an array of TLRs, whose signaling is collaborated with the B cell receptor signaling (91). Signaling from TLRs like TLR7 and TLR8 are well documented to augment the antibody and cytokine production from B cells (90). This TLR-mediated stimulation of B cells depicted increased expression of B7 costimulatory molecules and amplified survival as like B cell activation by CD40 (90). Activated B cells were reported to secrete multiple chemokines and cytokines after stimulation of TLR1/2, TLR7, and TLR9 (92). TLR-mediated signaling enhances cytokine secretion, promotes better antigen presentation from B cells along with overexpression of the costimulatory molecules, and, which sequentially augments the activation of helper T cells (91). Moreover, multiple reports demonstrated the activation of effector functions of B cells by TLR signaling, e.g., proliferation, antibody production, and immunoglobulin class switching (93–95). While B cells are known for their ample Toll-like receptor (TLR) expression and their critical role in humoral immunity and the adaptive immune response, their potential for TLR-mediated utilization in cancer immunotherapy remains relatively unexplored (91–93). Nevertheless, there have been a few instances where TLR-mediated activation of B cells has been applied in the context of cancer immunotherapy. B cells use TLRs to coordinate antibody responses during infection and autoimmune diseases, where the B cell receptors (BCR) and TLR7 or 9 are activated in response to self-antigens complexed with nucleic acids, such as RNA or DNA-containing immune complexes (96). This TLR-mediated stimulation can also be harnessed to generate tumor-specific antigen (TSA)-specific responses (44, 50). When high levels of antibodies are required for protection, be it infection or anti-tumor immunity, targeting TLRs on B cells can prove to be an effective strategy to enhance antibody production (97). The presence of tumor-specific antigens (TSAs) is essential for activating T cell and B cell immunity (98). Notably, B cells are the sole immune cells that consistently express TLR9 (97). Several studies have demonstrated that TLR9 agonists can induce significant anti-tumor immunity by activating B cells. TLR9 agonists endorse the differentiation of B cells into plasma cells and enhance antibody-dependent cellular cytotoxicity (ADCC) (99, 100). Brody et al. (101) reported clinically significant anti-B cell lymphoma responses following *in-situ* tumor vaccination with a TLR9 agonist. These studies underscore the efficacy and advantages of administering TLR agonists directly at the tumor site rather than systemically. TLR9 ligands like CpG-ODNs have shown great potential in stimulating B cell-mediated adaptive immunity (102–104). CpG-ODNs strongly induce B cell proliferation, activate plasmacytoid dendritic cells (pDCs) and monocyte maturation, stimulate natural killer (NK) cell activation, and trigger the production of inflammatory cytokines (102). B cell stimulation by CpG-ODNs increases their sensitivity to antigen stimulation and promotes their differentiation into antibody-secreting plasma cells, resulting in increased production of antigen-specific antibodies (103). TAC-001, an antibody-ODN conjugate consisting of a specialized TLR9 agonist (T-CpG) linked to an antibody against CD22 (a receptor restricted to B cells), is designed to deliver potent and targeted immune activation through systemic administration (104). *In vitro* stimulation of B cells with TAC-001 leads to increased expression of co-stimulatory molecules, immunoglobulin secretion, and cross-presentation, ultimately leading to T cell proliferation (104). TAC-001 has demonstrated efficient and durable single-agent anti-tumor activity in checkpoint inhibitor-resistant and refractory murine tumor models (104). Systemic administration of TAC-001 in mice has resulted in increased B cell infiltration, enhanced T cell effector functions, modulation of myeloid-derived suppressor cells (MDSCs), and a significant decrease in IL-10+ regulatory B cells within the tumor microenvironment (104). Intravenous administration of TAC-001 in monkeys has shown favorable tolerability, pharmacokinetics, and pharmacodynamic profiles (104). Additionally, TLR9 activation in B cells leads to the expression of co-stimulatory molecules, enhancing cross-presentation, and allowing for the activation and proliferation of T cells, as well as the secretion of chemokines, cytokines, and immunoglobulins (3). Among other TLR9 ligands, Lefitolimod (MGN1703) has been utilized in several preclinical studies to assess B cell-mediated immunity. Multiple studies have demonstrated that MGN1703 significantly activates both innate and adaptive immune cells, including B cells, and induces the secretion of various inflammatory cytokines (IL-6, IL-8, IFN-α, and IFN-γ) and chemokines (CD40, CD69, CD86, CD169, and IP-10) from activated immune cells (105, 106). The combination therapy of TLR3 agonist with a TLR9 agonist (CpG: 5’-cytosine-phosphate-guanine-3’) along with adoptive T cell transfer (ACT) has shown promise in increasing the abundance of various immune cell types, including B cells with CD4^+^ and CD8^+^ T cells, macrophages, neutrophils, and NK cells in tumor-draining lymph nodes (107). This combination therapy has augmented the elimination of murine melanoma cells and improved the survival of tumor-bearing mice, doubling their survival compared to untreated mice (107). Combination therapy involving TLR9 agonists and immune checkpoint inhibitors (ICIs) has also shown promising effects in clinical studies (108). For instance, Ribas et al. evaluated the safety and anti-tumor activity of co-treatment with intratumoral SD-101, a synthetic CpG oligonucleotide ligand for TLR9, and pembrolizumab in patients with melanoma (108). This combination therapy was well tolerated and improved overall survival, accompanied by a significant increase in B cells within the tumor microenvironment (TME), as well as other immune cell populations (108). These results indicate that combining SD-101 administration with PD-1 blockade potentially enhances clinical efficacy and reduces PD-1 blockade-related toxicity (108). Apart from TLR9, monophosphoryl lipid A (MPLA), a TLR4 ligand derived from the lipopolysaccharide (LPS) of Salmonella Minnesota, is used as an adjuvant in a prophylactic vaccine against human papillomavirus types 16 and 18, which are common causes of cervical cancer (109). As an adjuvant, MPLA enhances the antigen-presenting capabilities of macrophages and B cells, primes naive T cells, induces the maturation of dendritic cells, and stimulates antibody production (109).

### Effector T cells

The effector T cells carry out multiple functions of the immune responses, like cytotoxicity, helper, and regulatory (110). Diverse types of effector T-cells express different TLRs, which can consequently regulate associated T-cell functions and antitumor immune activities (111). TLR1, 2, 5, 7, and 8 mediated signaling is identified to activate the proliferation of CD4^+^ memory T-cells and upregulate accompanying cytokine secretion (112). For example, the presence of a higher amount of TLR5 agonists amplified CD4^+^ T-cell population and concomitant expression of the cytokine IL2 (112). Activation of multiple TLRs like TLR2, 3, and 9 in purified B6 expressing CD4+ T cells provides costimulatory signals aimed at T cell receptor (TCR) activation. NF-κB signaling through TLR2-mediated signaling in CD8^+^ T cells and TLR9 activation in CD4^+^ T cells inhibits apoptosis and promotes survival (113). In a similar study, activation of TLR7 along with TLR8 on CD4^+^ T-cells enhanced the secretion of IFNγ, IL-2, and IL-10; in addition to proliferation of T cells (114). Interestingly, the antitumor commotion of CD8^+^ T-cells was promoted via glucose-uptake-dependent MyD88 and AKT-mTOR pathway through activation of TLR7 (115). It is well established that stimulation of immune cells like NK cells, DCs, and Tregs can control the CD8^+^ effector T cell functions (51, 52, 82); similarly, multiple TLRs can control multiple functional characteristics of CD8^+^ T-cells directly inside the tumor microenvironment (116). Activation of TLR1/2 promotes the effector activity of CD8^+^ T cells in B16 melanoma cells both *in vivo* and *in vitro* through upregulation of perforin, Granzyme B, IFNγ and TNF-α (116). In addition, effector CD8^+^ T cell functions along with increased IFNγ expression as a functional coactivator was also reported to be regulated by activation of TLR3 (117). Besides, transgenic OT-1 (CD8^+^) T cells were stimulated through an antigen-independent manner after activation of TLR3, as measured by *in vitro* assays, leading to increased expression and robust expansion of immune-activation markers *in vivo* (118).

### Regulatory T cells

The immunosuppressive activity of both human and murine regulatory T-cells (Tregs) can be modulated by some of the TLRs, and it is documented in multiple reports. TLR4 activation on Tregs enhanced their viability and immunosuppressive commotion (112). Also, a slight ligand-mediated activation of TLR5 increased the Treg marker FOXP3 on CD4^+^CD25^+^ Treg cells and increases their immunosuppressive aptitude slightly (112). There is also a contrasting report of reverting Treg’s suppressive properties *in vivo* through the TLR8-MyD88-IRAK4 signaling cascade (119). There are some controversies regarding the reversion of suppressive activity of Tregs after TLR2 activation even though multiple reports depicted an increase in Treg proliferation after TLR2 activation (120, 121). For instance, activation of TLR2, inhibited the immunosuppressive activity of Tregs though it increased proliferation of Treg cells *in vivo* (122). In the tumor microenvironment (TME), activation of immunosuppressive cells like tolerogenic dendritic cells (DCs) and Treg cells is vital for establishing immunosuppression (123). Using antibodies to inhibit Treg cell function is an initial approach to enhance the effectiveness of cancer vaccines by reducing TME’s immunosuppressive effects and boosting effector T cell function (124). Consistently, administering a DC vaccine with the TLR4 agonist LPS resulted in a notable increase in NK cells and a significant reduction in Treg cells within the tumor microenvironment in an ovarian cancer mouse model (125). TLR ligands can also trigger Th1 inflammatory cytokines like IL-12, which facilitate the transition of CD4^+^ T cells from Th2 to Th1 subtype, boost CD8^+^ T cell responses, and suppress Treg cell function (126). TLR3 ligands were also reported to overturn the immunosuppressive TME towards anti-tumor immunity through modulation of the Treg cells (127, 128). TLR3 ligand poly A:U shifts the immunosuppressive tumor microenvironment towards anti-tumor immunity by altering the composition in favor of antigen-specific CD8^+^ granzyme B^+^ T cells, resulting in a lower Treg/CD8^+^ cell ratio (127). Salazar et al. reported that administration of poly-ICLC induced *in situ* vaccination in a rhabdomyosarcoma patient induced local tumor inflammation and a systemic immune response, leading to a significant reduction in a facial tumor (128). Their research revealed that tumor regression was a result of the activation of both local and systemic anti-cancer immunity triggered by intratumoral and intramuscular poly-ICLC injections. Their findings suggest that intramuscular poly-ICLC maintenance therapy contributes to a systemic anti-tumor immune response through the induction of chemokines, co-stimulatory molecules, inflammasome formation, and an increase in the Teff/Treg cell ratio (128). In addition to TLR3, TLR7 agonists also inhibit Treg cell function, and activate NK cells, promoting anti-cancer immune responses (87, 129). Topical imiquimod application in a melanoma mouse model reduces Treg cell-related chemokine mRNA expression and increases cytotoxic molecules like granzyme B and perforin within tumors (130). Imiquimod also decreases Tregs and boosts CD8^+^ cells in the tumor microenvironment (TME) (130). Intratumoral administration of SZU101(another TLR7 agonist) triggers a systemic anti-tumor response and alters the TME by increasing CD4^+^ and CD8^+^ cells while reducing Treg cells in a murine breast tumor model (131). Intraperitoneal injection of the TLR7 agonist resiquimod in mice with pancreatic ductal adenocarcinoma (PDAC) tumors reduces Tregs in the TME, enhances activation, infiltration, and cytotoxicity of CD8^+^ T cells, suppressing tumor growth and improving survival (65). Combination therapy of radiation and imiquimod decreases Treg cells and MDSCs while increasing CD4^+^ and CD8^+^ T cell recruitment in the TME, commencing systemic anti-cancer responses and potentially limiting metastasis ultimately leading to increased survival (132). Administrating imiquimod as adjuvant preceding HPV vaccination enhances intratumoral CD4^+^ and CD8^+^ T cell infiltration while reducing Treg cells in the TME (133). Importantly, the effectiveness of the vaccination correlates with pre-existing and post-treatment (with imiquimod) Treg cell levels (133). Combining PD-L1 blockade with resiquimod reduces tumor size, activates DCs, diminishes Treg cells, and boosts the CD8^+^ T cell/Treg cell ratio in the TME in mice tumor models (134). Furthermore, in the case of mice PDAC derived tumors, resiquimod elicited a robust immune response characterized by heightened immune complexity, reduced growth, enhanced infiltration of CD8^+^ T cells, and a lowered frequency of intratumoral CD4^+^CD25^+^FOXP3^+^ Treg cells (135). TLR9 ligands were also reported to modulate the TME via the suppression of Treg cells. CpG-A, the TLR9 ligand, induces IFNα and IFNβ production, promoting effector CD4^+^ T cell proliferation by counteracting Treg cell suppression (119). In a mouse tumor model, adoptive transfer of Treg cells after pretreatment with poly-G10 (a TLR8 ligand) enhances antitumor activity and reduces Treg cell suppression (119). Remarkably, this study also suggests that Treg cells express TLR9 and can recognize CpG DNA molecules (119). Furthermore, CpG ODNs reduce Treg cell population in the draining lymph node (136). Local administration of CpG enhances OX40 expression, a TNF receptor, on both effector T and Treg cells in the tumor microenvironment (TME) (137). PF-3512676, one of the earlier synthetic TLR9 agonists to treat melanoma patients, increases pDC and mDC frequency, along with the release of inflammatory cytokines, while markedly reducing Treg cell population in the sentinel lymph nodes (SLN) (138). In addition, SD-101 (TLR9 agonist) treatment also reduces numbers of Treg cells and T follicular helper cells within tumors (139).

## Application of TLR ligands/agonists in Cancer immunotherapy

With their ability to activate several innate immunity pathways, TLR ligands or agonists are considered compelling immunomodulators (44, 140, 141). Upon stimulation of TLRs by agonists, the downstream signaling also initiates enduring adaptive immune responses including cytotoxic NK cells, T-cells and maturation of DCs (140). Several TLR agonists demonstrated significant therapeutic efficacy against multiple ailments, including cancer (44, 141). Recent reports depicted improved efficacy of current immunotherapy approaches in cancer patients, such as cell-based immunotherapy combined with TLR agonists (19). TLR agonists are also known to sensitize cancer cells to conventional cancer therapies like radiation and chemotherapy (4, 140). Apart from combined therapy, TLR agonists are also administered as monotherapy in several malignancies (19). With growing information about several TLRs and involved TLR-agonists along with their downstream signaling pathways, several natural (resourced from microbes) or chemically synthesized TLR agonists, are being involved in cancer immunotherapy approaches (4, 140). But there are some early clinical setbacks in using TLR agonists as cancer therapeutics (142) because of the pro-tumorigenic nature of some TLRs in certain cancer types (44), and the activation of some of the TLRs led to an increase in tumor growth and metastasis (143). For this reason, it is necessary to determine the right tumor type and appropriate TLRs to be targeted and involved combinatorial approaches before treating any tumor with TLR-based immunotherapy. For the last two decades, TLR agonists have been used to stimulate and activate DCs in cancer immunotherapy (123). Moreover, in multiple vaccination strategies and immunotherapy approaches, TLRs can modify T-cell responses, which is revealed to be an excellent means to control and direct adaptive immunity (144, 145). One of the reasons for the growing popularity of TLR agonists for treating tumors is their aptitude to reinstate the activity of immunosuppressed DCs, which can be extremely useful for reversing the immunosuppressive atmosphere inside the TME (64, 65). TLR ligands have been widely utilized as adjuvants of anti-cancer vaccines, or in combination with other traditional standard of cares for cancer (19, 44). Some TLR agonists have shown great promise at the clinical scenarios (19, 44). In recent years, against poorly immunogenic tumors, synthetic TLR agonists performed markedly better as adjuvants of cancer vaccines by enhancing the Th1 or Th2-mediated immune response (146). In **Table 2**, we have listed all the current clinical trials where TLR agonists are used as adjuvant(s) to cancer vaccines (3, 4, 6, 7, 90).

**Table 2:** Clinical Trials that are testing TLR agonists as adjuvants of cancer vaccines.

Target	Molecule	Indication	Status	Vaccine or co-therapy	Phase	NCT number
TLR2	BCG	Melanoma	Unknown	In combination with cyclophosphamide, IL-2 and a melanoma specific vaccine	II	NCT00477906
TLR3	Ampligen	Colorectal carcinoma	Withdrawn	In combination with DC-based vaccination, interferon-α2b and celecoxib	II	NCT02615574
TLR3	Hiltonol (poly(I:C))	Breast carcinoma	Active, not recruiting	In combination with a peptide vaccine and durvalumab	I	NCT02826434
TLR3	Hiltonol	Breast carcinoma	Active, not recruiting	In combination with a peptide vaccine and pembrolizumab	I	NCT03362060
TLR3	Hiltonol	Gynecological tumors	Active, not recruiting	In combination with a DC-based vaccine, guadecitabine and atezolizumab	I/IIb	NCT03206047
TLR3	Hiltonol	Lung cancer	Active, not recruiting	In combination with a MUC1-vaccine	I	NCT03300817
TLR3	Hiltonol	Solid tumors	Completed	In combination with a personalized vaccine	I	NCT02721043
TLR3	Hiltonol	Solid tumors	Withdrawn	In combination with bevacizumab and a peptide vaccine	I	NCT02754362
TLR3	Hiltonol	Multiple myeloma	Active, not recruiting	In combination with a peptide vaccine and durvalumab ± lenalidomide	I	NCT02886065
TLR3	Hiltonol	Glioma	Active, not recruiting	In combination with a cancer cell lysate vaccine before and after or only after surgery	I	NCT02549833
TLR3	Hiltonol	Glioma	Active, not recruiting	In combination with a peptide vaccine ± varlilumab	I	NCT02924038
TLR3	Hiltonol	Glioma	Active, not recruiting	In combination with a peptide vaccine	I	NCT02960230
TLR4	G100	Solid tumors	Terminated	In combination with a NY-ESO-1-targeting vaccine	I	NCT02387125
TLR8	Imiquimod	Cervical intraepithelial lesions	Unknown	In combination with a DNA-based vaccine	n.a.	NCT03206138
TLR8	Imiquimod	Cervical intraepithelial lesions	Active, not recruiting	Alone or in combination with HPV vaccination	II	NCT02864147
TLR8	Imiquimod	Genital warts	Completed	In combination with a DNA-based vaccine	II	NCT03180684
TLR8	Imiquimod	Chronic lymphocytic lymphoma	Completed	In combination with a peptide-based vaccine and lenalidomide	II	NCT02802943
TLR8	Imiquimod	NSCLC	Unknown	In combination with a DRibble-based vaccine, DC/CIK cells and GMCSF	I	NCT03057340
TLR7/8	Resiquimod	Melanoma	Completed	Combined with a peptide-based vaccine	I	NCT01748747
TLR7/8	Resiquimod	Melanoma	Completed	Combined with a peptide-based vaccine	I	NCT00470379
TLR7/8	Resiquimod	Melanoma	Unknown	Combined with a peptide-based vaccine ± poly-ICLC	I/II	NCT02126579
TLR7/8	Resiquimod	Melanoma	Completed	Combined with a peptide-based vaccine	II	NCT00960752
TLR7/8	Resiquimod	NY-ESO-1+ tumors	Completed	Combined with a peptide-based vaccine	I	NCT00821652
TLR7/8	CV8102	Hepatocellular carcinoma	Completed	Combined with cyclophosphamide and a peptide-based vaccine	I/II	NCT03203005
TLR9	DUK-CPG- 001	Hematological neoplasms	Withdrawn	In combination with a DC vaccine	II	NCT02115126
TLR9	Vidutolimod	Chronic lymphocytic leukemia	Recruiting	Multipeptide vaccine, XS15	I	NCT04688385

Adapted from.

1. “Trial watch: Toll-like receptor ligands in cancer therapy”. By Le Naour J, and Kroemer G. 2023, Oncoimmunology. 12(1):2180237.

2. “Trial Watch: Toll-like receptor agonists in cancer immunotherapy”. By Smith M, García-Martínez E, Pitter MR, Fucikova J, Spisek R, Zitvogel L, et al., 2018, Oncoimmunology. 7(12):e1526250.

3. “Trial watch: intratumoral immunotherapy”. By Humeau J, Le Naour J, Galluzzi L, Kroemer G, and Pol JG. 2021, OncoImmunology. 10(1):1984677.

4. “Trial Watch: Immunostimulation with Toll-like receptor agonists in cancer therapy”. By Iribarren K, Bloy N, Buqué A, Cremer I, Eggermont A, Fridman WH, et al., 2016, Oncoimmunology. 5(3):e1088631.

5. “Trial Watch: experimental TLR7/TLR8 agonists for oncological indications”. By Frega G, Wu Q, Le Naour J, Vacchelli E, Galluzzi L, Kroemer G, et al., 2020,OncoImmunology. 9(1):1796002.

At the next section of this review, we are trying to present and discuss multiple clinical and preclinical pieces of evidence which demonstrates that TLR agonists can significantly improve the therapeutic outcome in different types of cancer, either in combined immunotherapy or as a cancer vaccine adjuvant.

## Application of TLR agonists as adjuvants of cancer vaccine

### TLR2/TLR4

Bacillus Calmette-Guerin (BCG) is the first successful TLR ligand/agonist approved for cancer treatment (147, 148). More than 40 years ago, BCG was first approved by the US Food and Drug Administration (FDA) to treat bladder cancer (147, 148). BCG wields its anti-cancer effect by the dual activation of TLR2, and TLR4 (147, 148). BCG as an adjuvant with whole cell vaccines also has been widely assessed in melanoma and colorectal cancer (CRC) (149, 150). In a randomized Phase III trial, Canvaxin, an allogeneic melanoma vaccine utilizing BCG as an adjuvant, failed to improve both overall and disease-free survival, despite showing promise in phase II (149). Interestingly, in the same trial with resected stage-III and stage-IV melanoma, monotherapy of BCG in patients demonstrated improved efficacy (149, 150). OncoVAX, an autologous colon cancer vaccine with BCG as an adjuvant, in a phase II study showed significant improvement in disease-free and overall survival (150, 151). Similarly, anti-tumor effect generated through activation of TLR2 and 4, by OM-174 (CXR-526), a lipid A (*Escherichia coli* origin) derivative, is currently being evaluated as a vaccine adjuvant for the treatment of melanoma in phase I/II trials, in addition to a phase I trial against solid tumors (152). The observed results from a few preclinical studies depict the increase of TNF-α, IFN-γ, and iNOS behind the therapeutic activity of OM-174 administration (152, 153). Another lipid A derivative, monophosphoryl lipid A (MPL), also an activator of TLR4, is also used in several vaccines as an adjuvant (153, 154). Stimuvax, the liposomal cancer vaccine against the MUC1 tumor antigen, uses MPL as an adjuvant (153). Stimuvax underwent phase III evaluation against advanced NSCLC but failed to add any marked therapeutic advantage (153). A cancer vaccine targeting the MAGE A3 tumor antigen utilizes a MPL containing special mixed adjuvant system (AS15, AS02b) (155). Other TLR4 activators like AS04 (MPL derivative, cervical cancer) and GLA-SE (lymphoma Merkel cell carcinoma) are also studied via preclinical and clinical studies (156–158). Meanwhile, lipoteichoic acids (LTA) from Gram-negative bacterial cell walls, responsible for the “endotoxin” of bacteria, can also act as an agonist of TLR2 receptors and trigger anti-tumor immune responses (11, 14).

### TLR3

The activation of TLR3-mediated signaling by sense double-stranded RNA was first discovered by Alexopoulou et al. (159). Currently, the synthetic polynucleotide polyinosinic-polycytidylic acid or poly(I:C) is being used as a TLR3 agonist, and is known as a powerful activator of the innate immune responses (160). Poly(I:C) promoted the activation of DCs and subsequently enhanced antigen presentation for CD8^+^ cytotoxic T cells (161). Additionally, after stimulation via poly(I:C), DCs can indirectly activate NK cells and T-cells, generating robust antitumor immune responses, and for this reason poly(I:C) is often employed in cancer vaccines (162). Because of the associated toxic effects and hasty degradation of poly(I:C) in the body, several stable derivatives or variants of poly(I:C) are established through experimental studies (163, 164). For example, Hiltonol or poly-ICLC is stabilized through the addition of poly-lysine and was assessed in several clinical trials involving different types of solid tumors (165, 166). Though these clinical studies didn’t demonstrate significant anti-tumor efficacy but still it managed to be physiologically safe without any adverse side effects (165, 166). Additionally, rintatolimod or poly(I:C_12_U) (Ampligen), another poly(I:C) derivative, is stabilized by the substitution of cytidine with uridine and is approved for the treatment of pancreatic, triple-negative breast cancer, and brain tumors in combinatorial therapy with some vaccines demonstrated substantial efficacy (167–169). Poly(I:C) and all of its derivatives induce maturation of DCs, as well as intensify the expression of Th1-related cytokine, and currently evaluated in multiple clinical trials as potent vaccine adjuvants (170). In addition, poly-ICLC was moderately successful as combinatorial therapy with peptide or DC vaccines in various advanced malignancies, including malignant glioma (136).

### TLR5

TLR5 is activated by bacterial flagellum protein flagellin, and flagellin derivatives are evaluated for anticancer efficacy (171–173). The clinical efficacy of CBLB502 (natural flagellin/entolimod derived from natural *Salmonella* flagellin) is evaluated against squamous cell head and neck cancer and solid tumors in phase I clinical trials (174). Mobilan, a recombinant nonreplicating adenovirus encoding flagellin which is commercially available as M-VM3 is also has been studied for anti-cancer effects in prostate cancer (175). Treatment of breast cancer cells with the TLR5 agonist flagellin also reported to suppress cell proliferation and inhibiting anchorage-independent tumor growth (171). In another interesting *in vivo* study, the researcher demonstrated contrasting effects on tumor growth after TLR5 activation by flagellin. Activation of TLR5 by flagellin reduces the growth of strongly immunogenic tumors, but it failed to do the same for weakly immunogenic variants (172). These conflicting results were caused by the disproportionation between IFN-γ:IL-4 ratio and the concomitant number of CD4^+^CD25^+^ T regulatory cells (172). Also in the same study, early combinatory treatment of flagellin and CpG-containing oligodeoxynucleotides (CpG ODNs) completely inhibited tumor growth (172).

### TLR 7/8

Among all the TLRs, ligands or agonists of TLR7 and 8 have shown the most promising immunomodulatory and anticancer effects, and many of them transitioned to the clinic (176). TLR7 and 8, both recognize ssRNA as ligands, and this property has been exploited to synthesize several types of TLR7/8 agonists that could achieve stimulation of these receptors simultaneously (176). Based on chemical structures, TLR7/8 agonists are organized into guanosine and adenosine analogs or imidazoquinoline derivatives with modified RNA sequences (176, 177). Dual agonists of TLR7/TLR8, resiquimod (R-848), and loxoribine (178) failed to enter phase III trials because they lacked significant efficacies (179). Initial studies attributed the lack of local immune activation by resiquimod to its property of solubility in body fluid and subsequent dispersion from the injection site (178). Resiquimod was thereafter administered as a dermal cream to counteract this problem (180). Resiquimod induces the expression of TNF-α, IFN-α, and other proinflammatory cytokines, via the initiation of the TLR7-MyD88-dependent pathway (22). Imiquimod, another imidazoquinoline marketed as Aldara (5% imiquimod cream), was approved by the European Medicines Agency and FDA in 1997 for treating human papillomavirus (HPV) induced genital warts (181). Later in 2004, imiquimod was also approved as therapeutics of primary skin malignancies like superficial basal cell carcinoma and premalignant actinic keratosis (182). Topical ointment of imiquimod is also used for the treatment of other local cutaneous tumors, including melanoma, and interestingly imiquimod exerts an impressive over 85% success rate while treating lentigo maligna melanoma (183, 184). Imiquimod also demonstrated significant efficacy in combinatorial therapies with other traditional anticancer therapies like chemotherapy or radiotherapy in multiple cancer types (180). Imiquimod has also shown promise in developing DC-based vaccines (6, 185). Imiquimod promoted the stimulation and maturation of immature DCs and their consequent migration to draining lymph nodes in cancer patients (185). Topical imiquimod ointment amplified the immunogenicity of the peptide vaccine for melanoma (185). In patients with resected melanoma, imiquimod as an adjuvant also augmented the immunogenicity of the NY-ESO-1 peptide vaccine, recruiting and activating pDCs and mDCs subcutaneously and in inflammatory infiltrates (6). In contrast to imiquimod’s topical application, 852A (a TLR7 agonist) and VTX-2337 (a TLR8 agonist) are dispensed systemically, and their efficacy are under evaluation through multiple phase I/II clinical trials involving malignant breast, ovarian, endometrial, cervical, and head and neck cancers (186). 852A activates APCs and stimulate NK cells with increased secretion of IFN-α from plasmacytoid DCs in cancer patients (179, 187). In a phase II trial, 852A demonstrated safety and systemic immune activation in metastatic melanoma patients who had failed chemotherapy, leading to prolonged disease stabilization (188). While treating patients suffering from chronic hepatitis C virus (HCV) infection and cancer, ANA773- the orally administered TLR7/8 agonist, induced IFN-α, activated NK cells, and reduced serum HCV RNA levels (189). Lastly, 3M-052 (a lipid-modified imidazoquinoline derivative), was assessed as a cancer vaccine adjuvant demonstrating marked synergistic efficacy in tandem with checkpoint-blocking antibodies for CTLA4 and PDL-1 (190). This result highlighted the potential on the aspect of utilizing TLR7/8 agonists in combinatorial therapies with other immunotherapeutic agents like immune-checkpoint inhibitors (ICIs).

### TLR9

Unmethylated cytidine phosphate guanosine (CpG) and oligonucleotides (CpG ODNs) are the main ligands or agonists for TLR9 receptors (191). Several CpG oligodeoxynucleotides are verified for their anticancer effects both *in vitro* and *in vivo* models of multiple cancers (192–194) and clinical trials (195). Some of the significant examples of TLR9 agonists undergoing clinical trials are IMO2055 (a CpG ODN-based oligonucleotide tested in advanced NSCLC), dSLIM (two single-stranded oligodeoxynucleotide loops connected with double-stranded oligodeoxynucleotide stem, currently tested in advanced colorectal cancer), MGN1703 (a natural DNA molecule assessed in small cell lung cancer and advanced solid tumors), CpG-7909 (a single-stranded CpG ODN, currently evaluated in melanoma, renal cell carcinoma, non-Hodgkin’s lymphoma, glioblastoma, cutaneous T cell lymphoma, and NSCLC), KSK-CpG (phosphorothioated derivative of CpG ODNs, being evaluated in melanoma), SD-101 (tested in follicular lymphoma), ODN M362 (tested in hepatocarcinoma), and CpG-1826 (demonstrated amplified antitumor effect in glioma xenografts) (106, 191, 196, 197). Even after promising preclinical studies, the administration of IMO2055 in combination with platinum-based drugs against recurrent and metastatic head and neck cancer, raised some safety issues in a phase II trial (142). Similar safety concerns were raised against CPG7909, in a phase III trial involving NSCLC (142). CpG ODNs blended (emulsified) with Montanide ISA 51, being used as an adjuvant with vaccines targeting cancer-testis antigens, demonstrated promising results by promoting pDC-mediated infiltration of lymphocytes at the site of vaccination (198). Therefore, the application of CpG ODN as an adjuvant of cancer vaccines or intratumoral injection could be a potential opportunity to direct effector lymphocyte-mediated response.

### TLR ligands as adjuvants in autologous MBTA vaccine immunotherapy

Till now in the previous sections, we discussed the classification, the immunomodulatory functions of TLRs, and the administration of TLR agonists in immunotherapeutic strategies and cancer vaccines. We concisely described how different TLR agonists are employed as adjuvants to cancer vaccines in several preclinical and clinical studies. Most of the current immunotherapy trends are heavily oriented toward the onset of adaptive immunity via immunomodulators like TLR agonists. Here we present a new immunotherapeutic approach developed by our research group based on autologous tumor neoantigens and TLR agonists that are proficient in triggering both the innate and adaptive immune responses (199, 200). It’s an autologous vaccine strategy that leverages the PRR assets of TLRs as well as fungal polysaccharides, and it has been tested in diverse types of mouse tumor models (11, 14, 199–201).

**Figure 2:**
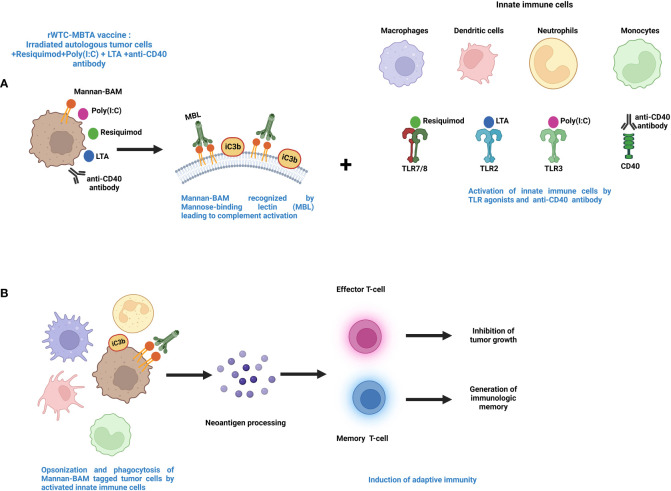
Mechanism of action for MBTA vaccine therapy. **(A)**. The MBTA vaccine consists of mannan-BAM tagged irradiated cancer cells mixed with TLR ligands resiquimod, poly(I:C), and LTA along with anti-CD40 antibody. The polysaccharide mannan is chemically linked with the hydrophobic lipid tail biocompatible anchor for membrane (BAM). The hydrophobic lipid tail enables the attachment of mannan to the plasma membrane of irradiated tumor cells. Mannan-BAM acts as a PAMP and exploits the pattern recognition properties of Mannose-binding lectin (MBL). This recognition of Mannan-BAM by MBL culminates into the activation of the lectin pathway of complement activation through the proteolytic cleavage of complement protein C3, and iC3b, the inactive cleaved form of C3 initiate the opsonization of the tumor cells. Concurrently, the three TLR ligands (resiquimod/R-848, poly(I:C), LTA) and anti-CD40-antibody act as adjuvants facilitate recruitment of the innate immune cells like macrophages, dendritic cells, neutrophils, and monocytes into the tumor (199–202). **(B)**. The TLR agonists activate the innate immune cells with augmented expression of inflammatory cytokines and chemokines that endorse maturation of APCs. The activated APCs opsonize and phagocytose the tumor cells and process tumor neoantigens. These activated APCs further internalize tumor antigens and display them to T cells in lymph nodes. This leads to the initiation of adaptive immune cells like effector T cells along with generation of immunologic memory through memory T cells (199–202).

This vaccine is called rWTC-MBTA, comprising of irradiated autologous tumor cells (rWTC) mixed with mannan-BAM, TLR agonists, and anti-CD40 antibody (MBTA) (199–202). This vaccination strategy was initiated with the utilization of TLR agonists accompanied by concurrent labeling of tumor cells with phagocytosis-inducing ligands leading to enhanced recognition of the tumor cells by the different immune cells (203, 204). Mannan, a branched polysaccharide from the yeast *Saccharomyces cerevisiae*, is affixed to the cancer cell membranes through linkage with the hydrophobic BAM (Biocompatible Anchor for Membrane) anchor and serves as a PAMP, which in turn is recognized by the mannan-binding lectin complex (MBL) (11, 14) (**Figure 2**). Recognition of mannan-BAM by MBL activates the lectin pathway of complement activation and ultimately leading to iC3b-mediated opsonization and phagocytosis of tumor cells (11, 14, 201). To augment the vaccine-induced innate immune responses through the initiation of multiple inflammatory pathways, along with mannan-BAM, we use TLR ligands lipoteichoic acid (LTA)(agonist of TLR2), polyinosinic-polycytidylic acid (poly(I:C))(agonist of TLR3), and resiquimod (R-848)(agonist of TLR7/8) (11, 199–201) (**Figure 2**). Several previous reports from our group demonstrated the applicability of TLR ligands as adjuvants in rWTC-MBTA vaccines (11, 199–201). This vaccine strategy plays on stimulating the immune system on various stages, starting from primary activation of innate immunity trailed by concomitant activation of adaptive immunity (11, 14, 201). Each TLR ligands as adjuvants in the vaccine as unique function. The strongly immunogenic LTA, which is sourced from the Gram-Positive bacteria *Bacillus subtilis*, stimulates the TLR2-mediated inflammatory pathway, resulting in elevated secretion of TNF-alpha and heightened inflammatory response (205, 206). Poly(I:C) triggers TLR3-mediated signaling, leading to activation of antigen-presenting cells (APCs) and activates tumor associated macrophages (206–208). R-848 or resiquimod induces the activation of innate immune cells and the promotes Th1 cell-mediated immune responses (209, 210). In addition, anti-CD40 monoclonal antibodies in the vaccine preparation binds with the CD40L ligand to activate the CD4^+^ T lymphocytes, permitting the dendritic cells to mediate the adaptive immune responses (199, 200) (**Figure 2**). To prepare rWTC-MBTA-Vax, (i) autologous or syngeneic cancer cells are irradiated so that it remains alive but non-replicative, (ii) irradiated cells are combined with mannan-BAM and TLR agonists, along with anti-CD40 antibody, to produce the effective vaccine, and (iii) the prepared rWTC-MBTA is injected peripherally over four weeks to propagate a tumor-specific immune response to inhibit tumor or cancerous growth, metastasis, and prevent recurrence (199, 200).

One of the foremost advantages of the MBTA vaccines or rWTC-MBTA is that it can affect the immunogenic status of the TME to facilitate the outcome of the immunotherapy. The “cold” TME of some solid tumors is a major barrier to cancer immunotherapy (211–213). The immunogenically cold TME status is associated with a lack of inflammatory T-cell infiltration and lower neoantigen presentation (214). TLR agonists can promote Th1(T helper) mediated inflammatory responses and activate APCs in the TME, facilitating tumor infiltration as well as improved functioning of the effector immune cells, like CD8^+^ T cells and NK cells (212). When used as adjuvants, the TLR agonists augment the antigen presentation capacity of APCs and initiate the expressions of Th1 inflammatory cytokines along with increasing the expression of several co-stimulatory factors (126, 212, 215). The Th1 family of inflammatory cytokines endorse the switching of CD4^+^ T cells from Th2 subtype to Th1 subtype, increase CD8^+^ effector T-cell responses, and impede the immunosuppressive activity of Treg cells (126). The MBTA vaccine approach manipulates the TME, by the application of TLR agonists to turn the cold TME to hot, and it was evident by the efficacy of rWTC-MBTA on curbing immunogenically cold tumors like glioblastoma multiforme (GBM) (200) or triple-negative breast cancer (202).

Previous investigations involving intratumoral MBTA injections sustained a profound antitumor response, but patients are prone to secondary inflammatory damage or mass effect due to the *in situ* delivery of the vaccine (151, 216). Earlier studies of the MBTA anti-cancer therapeutics involved a unique combination of two different types of PAMPs, mannan-BAM serving as a tag for phagocytosis and soluble TLR agonist ligands as triggers of innate immunity (11, 14). This unique amalgamation of mannan-BAM and TLR agonists generates a robust infiltration of inflammatory cells toward the tumor. This led to reduction of tumor burden and even, in some experimental mice models, complete remission of the tumor (11, 14, 203, 204). In our preceding study, in a colon carcinoma preclinical mouse model, compared to control or injecting irradiated whole tumor cells alone, we found that subcutaneous injection of the rWTC-MBTA vaccine (irradiated whole tumor cells mixed with MBTA) caused a substantial decrease in tumor volumes and improved overall survival (199). In one of our recently published manuscripts, we demonstrated that the rWTC-MBTA vaccine effectively inhibited the metastasis and impeded the growth of tumors in animal models of both breast cancer and melanoma (202). Additionally, in a therapeutically mimicking postoperative model of breast cancer, it prevented the metastasis of residual tumors and extended the survival (202). Our results also demonstrated that the rWTC-MBTA vaccine effectively prevented the growth of autologous tumors but rendered ineffective against allogeneic tumors (202). Mechanistic studies regarding the rWTC-MBTA vaccination revealed enhanced activation of APCs, heightened CD4^+^ and CD8^+^ T-cell mediated response, generation of immune memory along with tumor specific cytotoxicity (202). Moreover, we also proved rWTC-MBTA vaccine efficacy was T-cell dependent through T-cell depletion assay (202). With the surmounting preclinical evidence, we want to translate rWTC-MBTA to the clinic for further investigations, and altogether the efficacy of rWTC-MBTA is dependent on TLR agonists, and this can direct toward a new track of cancer immunotherapy.

## Conclusions and future perspectives

TLR activation sparks immune responses against pathogens, making TLR agonists promising cancer immunotherapy. Targeting TLRs, alone or combined with other methods, offers a potential pathway to enhance the immune system and eradicate cancer cells. TLRs are crucial components of the immune system, playing a significant role in both innate and adaptive immunity. It’s exciting to know that there are currently various TLR agonists being evaluated in both preclinical and clinical settings worldwide. However, there are some foremost expected roadblocks with the implementation of TLR agonists as therapeutic options. Toll-like receptors in cancer have a dual role; on one side, they activate innate immunity, recruiting immune cells to eliminate invasive pathogens like tumor cells, but they can also contribute to chronic inflammation, driven by TLRs, resulting in anti-apoptotic effects through NF-κB and promoting tumor growth (217, 218). As TLRs regulate the stimulation of several immune cells of the human body, any improper tuning of the TLR agonists can trigger autoimmune diseases. There are also chances of the onset of chronic inflammatory side effects through uncalled activation of cytokines. Multiple examples of inflammatory side effects exist while using TLR ligands in immunotherapy (142, 217, 219, 220). Therefore, choosing the right TLR agonist for the treatment of a specific type of cancer is a vital issue to diminish the chances of post-therapeutic complications.

Both pre-clinical and clinical findings suggest that combining TLR agonists with antigens, immune modulators, or other treatments can enhance their effectiveness (19, 221). Therapies like chemotherapy or phototherapy release tumor antigens and cellular factors from dying cells, further activating dendritic cells and promoting cross-presentation to T cells (221). Current research highlights the potential of TLR-targeted drugs in cancer treatment. However, it’s crucial to acknowledge that factors like tumor characteristics and the microenvironment can impact the clinical success of TLR-targeting immunotherapies. These variables should be carefully addressed, especially in preclinical animal studies. Recent data suggests that combining TLR antagonists with other immunotherapy approaches, like checkpoint inhibitors and cell-based treatments, could improve overall immunotherapy effectiveness (19). Current trends of using TLR agonists in clinical trials project them as a compelling booster of immune responses in combination with cancer vaccines or other therapeutic approaches, suggesting a more promising strategy (**Table 2**). Another challenge is the difficulty of translating many TLR agonists from animal studies to human applications due to significant species-specific differences in TLRs (97). For instance, murine TLR8 reacts differently to imiquimod and R848 compared to human TLR8 (97). This highlights the importance of assessing potential TLR agonists in appropriate animal models and considering species-specific variations when interpreting results. Despite the potential of TLR agonists to activate the immune system for anti-tumor effects, they face persistent limitations. For instance, small molecule TLR ligands often fail to accumulate adequately in lymph nodes to activate immune cells effectively, leading to drug resistance. Rapid dispersion of TLR ligands can also trigger the production of immunosuppressive factors and undesirable immune responses. Additionally, these agonists/ligands have a short *in vivo* lifespan, especially endosomal TLR ligands, which are vulnerable to nucleases (222). As a result, innovative and efficient delivery platforms like dendrimers, stimuli‐responsive polymeric particles, liposomes, hydrogels, lipoprotein‐based scaffolds, and complexes have been devised to address these challenges (222).

Here in this review article, we presented the immense therapeutic potential, background aspects, and key investigations of using TLR ligands or agonists as an emerging component of immunotherapy. We also review the current landscape of using TLR agonists in cancer immunotherapy, including ongoing clinical trials and their drawbacks. Moreover, we explored how TLR agonists can induce diverse components of the immune system and how they are or could be applied as adjuvants of cancer vaccines. Last but not the least, our research group implemented TLR agonists as adjuvants augmented the immunogenicity of the rWTC-MBTA whole-cell autologous cancer vaccine. By continuing further studies, we have confidence in TLR agonist-based immunotherapy, in combination with other conventional therapies like surgery, chemotherapy, and radiotherapy, can shift the much-needed paradigm in the treatment of cancer and confirm an improved quality of life (QoL) for cancer patients.

## Author contributions

SC did the literature survey and wrote the article. JY, HW, MS, YZ, and XS contributed valuable inputs on the scientific contents. ZZ planned the sections of the article with SC. and gave scientific inputs. All authors have revised the manuscript and approved its submission.
